# Scoping review on the relationship between microRNAs (miRNAs) and short sleep disorder or insomnia with short sleep duration

**DOI:** 10.1016/j.sleepx.2026.100184

**Published:** 2026-04-02

**Authors:** Susana Perdigoto, Miguel Meira e Cruz, Manuel Remesal, Mauro Scala, María del Rocío González Soltero, Clara Azpeleta Noriega, Miguel de Pedro

**Affiliations:** a Universidad Europea de Madrid, Faculty of Biomedical and Health Sciences, Madrid, Spain; b Orofacial Pain, Oral Pathology and Dental Sleep Medicine Research Group. University Dental Clinics. Universidad Europea de Madrid, Madrid, Spain; c Sleep Unit, Cardiovascular Centre of the University of Lisbon (CCUL@RISE), Lisbon School of Medicine, Lisbon, Portugal; d European Sleep Centre, Lisbon, Portugal; e Department of Biomedicine and Dentistry, Faculty of Biomedical Sciences and Sports, Universidad Europea de Andalucía, Málaga, Spain; f School of Medicine, Complutense University of Madrid (UCM), Madrid, Spain; g Health Research Institute Hospital 12 de Octubre (imas12), Madrid, Spain; h Molecular Microbiology Group, Health Research Institute of the University Hospital La Paz (IdiPAZ), Hospital Universitario La Paz, Madrid, Spain; i Universidad Europea de Madrid. Faculty of Medicine, Health, and Sports. Department of Medicine, Madrid, Spain

## Abstract

**Background:**

MicroRNA (miRNA) stands for a class of small, non-coding RNA molecules, typically forming 20 to 25 nucleotides in length, which play a pivotal role in the regulation of gene expression in eukaryotic cells. These molecules are integral to the post-transcriptional regulation of target messenger RNA (mRNA), which they achieve primarily through binding to complementary sequences within the 3′ untranslated region (UTR) of the mRNA. miRNAs exert their regulatory effects by binding to their target mRNA, leading to two primary outcomes: mRNA degradation or inhibition of translation. This functionality positions miRNAs as crucial modulators of a variety of biological processes, including cell growth, differentiation, apoptosis, and responses to environmental stress. Furthermore, dysregulation of miRNA expression is associated with pathological conditions, including cancer, cardiovascular disease, and neurological disorders. They are associated with sleep regulation and are helpful in diagnosing diseases, including sleep disturbances. This scoping review aims to summarize existing literature in patients aged 18 to 65 with a main diagnosis of Short Sleep Disorder (SSD), and Insomnia with Short Sleep Duration (ISOSS) and its relationship with miRNAs. The objective of this study is to search for existing evidence on the composition (presence, diversity, and relative abundance) of miRNAs in individuals with and without sleep disturbances, and to know whether the cardiometabolic comorbidities associated with these pathologies imply miRNA alterations.

**Methods:**

This scoping review was performed according to the PRISMA-ScR checklist and the systematic literature search was conducted using Medline, Web of Science, and CINAHL, until June 2025.

**Results:**

Seven cross-sectional studies form the basis of this ScR. The miRNAs of particular interest include miR-182-5p, miR-4433b-3p, which are implicated in inflammatory pathways, and appear to be downregulated in conditions associated with sleep deprivation, thereby contributing to an exacerbation of pro-inflammatory responses that can lead to cardiovascular dysfunction. Also, miR-619-5p, linked to stress responses and cognitive and emotional health, miR-33a, miR-181d, implicated in lipid metabolism and neuroinflammation, miR-132-3p linked to increased risk of depression and cognitive decline and miR-125a, miR-126, and miR-146a, critical regulators of diverse biological processes, particularly in the context of inflammation and cardiovascular health.

**Conclusions:**

The existing literature sets up a foundational understanding of the relationship between miRNAs, insomnia (IS), SSD and ISOSS. Although the current evidence base is limited, dysregulation of specific miRNAs may play a role in the pathophysiology of IS, SSD and ISOSS; however, larger and methodologically standardized studies are required.

## Highlights

• Seven studies evaluated miRNA profiles in SSD and ISOSS
• miR-182-5p, miR-4433b-3p and miR-619-5p were recurrent findings
• Cardiometabolic links were direct in only three studies
• High methodological heterogeneity limits comparability
• No unified miRNA signature for SSD/ISOSS can be defined

## 1 Introduction

Insomnia (IS) is a prevalent disorder characterised by persistent and recurrent difficulties in initiating, maintaining, or returning to sleep [1]. One of the hallmark symptoms of IS is the experience of unrefreshing sleep [2], which significantly affects the general well-being of the individual [3]. This condition often co-occurs with various psychiatric disorders [4], notably depression [5], as well as somatic disorders such as autoimmune diseases [6].

Recent studies have highlighted a specific subset of IS, termed short-duration target sleep insomnia (ISOSS), which has been associated with cardiometabolic disturbances, including hypertension and diabetes [[7], [8], [9], [10]]. This association is particularly pronounced when comparing people experiencing ISOSS with those who achieve a suitable duration of sleep.

IS is a multifaceted, stress-related polygenic disorder [11]. The risk factors associated with IS have been causally linked to a variety of mental and medical disorders, suggesting a complex interrelationship likely arising from an interplay of genetic predispositions and environmental influences [12], with stress-related sleep reactivity emerging as a critical trait.

Furthermore, evidence suggests that IS, short sleep disorder (SSD) and ISOSS, and their interplay, are linked to epigenetic mechanisms that contribute to the acceleration of biological aging [13].

Although the body of research on the epigenetics of IS remains limited, the available studies provide a compelling framework for elucidating the long-lasting effects resulting from the interaction of genetic and environmental factors, particularly in the context of stress and its impact on neurobiology. For example, Xiao Li and coworkers have shown that sleep deprivation or melatonin reductions directly lead to decreased miR-182-5p synthesis, resulting in the accumulation of reactive oxygen species [14]. The implications of habitual short sleep duration are profound, correlating with heightened morbidity and mortality rates, primarily attributable to increased inflammatory burden and endothelial dysfunction.

In a related study, Su-Jin Baek and coworkers identified two miRNAs, miR-4433b-3p and miR-619-5p, in patients suffering from short sleep duration, revealing that the target genes of these miRNAs were associated with the regulation of circadian rhythms and inflammatory pathways [15]. miRNAs serve as epigenetic modulators, mediating phenotypic expressions through the regulation of specific target mRNAs [16]. The phenotypic consequences driven by these miRNAs underscore the necessity for establishing direct connections between objective gene regulation and observable in vivo phenotypes [17] to comprehensively understand the roles of miRNAs in the pathogenesis of various disorders [[18], [19], [20], [21], [22]].

Given their regulatory capabilities, miRNAs are promising candidates to advance our understanding of the pathophysiological mechanisms underlying IS. This scoping review (ScR) aims to comprehensively summarize the existing literature on the composition—including the presence, diversity, and relative abundance— of miRNAs in individuals diagnosed with SSD or ISOSS. Additionally, this review tries to investigate whether there is substantial evidence linking cardiometabolic comorbidities in patients with ISOSS and SSD to alterations in miRNA profiles.

Specifically, we will systematically map the existing literature that addresses the following primary questions:

1. How is literature addressing the differencial expression and regulation by miRNA in SSD or in ISOSS?
2. Does the composition (presence, diversity, and relative abundance) of miRNA in SSD or ISOSS differ from that of miRNA in individuals without sleep disturbances or those with insomnia but without a short duration of sleep?
3. Is there sufficient evidence that cardiometabolic comorbidities associated with patients with IS, SSD and ISOSS imply miRNA alterations?

Secondary questions are as follows:

1. Is there a potential association between specific miRNA and the severity of SSD or ISOSS symptoms?
2. Is there sufficient evidence that SSD or ISOSS treatment could affect miRNA quantity or quality over time?

## 2 Methods

This ScR was conducted according to the Joanna Briggs Institute (JBI) methodology for ScRs [23]. PRISMA (Preferred Reporting Items for Systematic Reviews and Metanalyses) guidelines were followed using the PRISMA extension for ScR (PRISMA-ScR) checklist [23]. The protocol was registered at the Open Science Framework (OSF): https://osfhttps://osf.io/vfm6t/.

To provide a structured approach to ensure the ScR is systematic, the following items were addressed [24,25]:

• To identify the research question.
• To determine relevant studies, inclusion criteria, study types, population, and contexts.
• To select studies by screening them with inclusion and exclusion criteria.
• To extract data using a standardized charting form.
• To summarize and report results, organize and present findings.
• To consult experts to enhance the review process and findings.
• To analyze evidence and summarize implications.
• To present the results.

### 2.1 Search strategy

A systematic search was conducted in June 2025 in the following electronic databases: Medline Complete, Web of Science (WOS), and the Cumulative Index to Nursing and Allied Health Literature (CINAHL).

Search strategies were tailored to the syntax and indexing system of each database. Controlled vocabulary terms (e.g., MeSH in Medline) and free-text keywords related to microRNAs (miRNAs) and short sleep phenotypes (SSD or ISOSS) were combined to maximise sensitivity. Search terms included “microRNA,” “miRNA,” “exosomal miRNA,” “circulating cell-free miRNA,” and “epigenomics.” For broader research questions, all relevant terms were combined within the same search strategy.

To ensure comprehensive coverage, the reference lists of relevant reviews and included studies were manually screened for additional eligible records. An updated search was conducted immediately prior to the final analysis to identify newly published studies. No restrictions were applied regarding publication date or study duration.

### 2.2 Operational definitions

In this scoping review, *short sleep duration* (SSD) was operationalized as sleep duration below commonly used thresholds reported in the included literature. When objective measures were available (actigraphy or polysomnography), SSD was defined according to the study's prespecified cut-off (typically <6 h/night). When only subjective measures were available (e.g., PSQI-derived sleep duration or self-reported habitual sleep), SSD was defined according to the study's prespecified cut-off (often <7 h/night).

*Insomnia with objective short sleep duration* (ISOSS) was defined as insomnia/insomnia symptoms combined with objectively measured short sleep duration (actigraphy or PSG), as reported by the included studies.

Given heterogeneity across definitions and measurement approaches, we extracted and reported SSD/ISOSS cut-offs exactly as defined by each study and charted results stratified by measurement modality (objective vs subjective).

### 2.3 Eligibility criteria

Articles included accomplished with:

1) Involving adult patients aged 18-65 with IS, SSD, or ISOSS. Sleep quality studies accessed by questionnaires, whether they are Insomnia Severity Index (ISI), Pittsburg Sleep Quality Index (PSQI) or any other validated questionnaire, actigraphy or polysomnography types I, II, III or IV.
2) Examining circulating exosomes or circulating miRNAs isolated from plasma, saliva, or any other human fluid, and may or may not include a study of the functional analysis of the global miRNA.
3) Analyzing miRNAs via small RNA sequencing, next-generation sequencing (NGS) or two-step quantitative RT-PCR. Receiver operating characteristic (ROC) analysis used to validate the identified miRNAs. In silico analysis or other analysis could be used to identify the target genes.
4) May contain biochemical measures before and after sleep deprivation of the systemic (plasma-derived) redox-metabolism including the major antioxidant, glutathione as well as DNA methylation levels.
5) Peer-reviewed, full-text studies published in English, such as interventional or observational study designs such as cross-sectional, case-control, and cohort studies.

No further restrictions were applied, such as geographic location, clinical setting (e.g., hospitals, outpatient, and inpatient rehabilitation settings), gender, or ethnicity of participants.

Studies were excluded when the primary focus was a non-sleep disorder (e.g., neurological, oncologic, infectious conditions) and sleep phenotyping (insomnia/SSD/ISOSS) was absent or secondary. Studies including cardiometabolic comorbidities (e.g., obesity, hypertension, diabetes) were eligible provided that insomnia/short sleep phenotyping remained a primary exposure/outcome and miRNAs were quantified in human biofluids. This decision was made to preserve alignment with our objective of mapping miRNA patterns linked to short-sleep insomnia phenotypes and their cardiometabolic context.

Conference abstracts and newsletters were also excluded as they were unlikely to contain the necessary elements for data extraction. Animal studies or those involving children or adolescents were not considered.

### 2.4 Study selection

Following the electronic search, all identified records were imported into Endnote 2025 and uploaded to the online software Covidence (https://www.covidence.org/), where Covidence removed the duplicates automatically. Two independent reviewers (S.*P. and* C.A.) screened titles and abstracts of the records according to the established inclusion and exclusion criteria. Selected citations were subsequently assessed in full text, with reasons for exclusion meticulously documented in Covidence. In instances where the full text was unavailable online, one researcher (S.P.) contacted the authors directly and requested articles through the interlibrary loan service of the European University (Madrid). Studies lacking peer-reviewed evidence or those for which the authors did not respond were excluded from the review.

Inconsistencies in screening decisions were resolved through consultations with another reviewer (M.R.G.). In instances of disagreement among the reviewers, four other reviewers (M.S., M.D.P., M.M., and M.R.) contributed to the resolution process. The search results, alongside the study inclusion process, were meticulously documented in the forthcoming ScR, which will feature a PRISMA flow diagram to delineate the methodological approach undertaken.

### 2.5 Data extraction and presentation of results

Two independent reviewers, S.*P. and* M.D.P., used Covidence to extract data from papers in the scoping review. They collected demographics, sample characteristics, miRNA data, short sleep disorder symptoms and measurement methods, associated cardiometabolic disorders, confounding variables (e.g., pharmacotherapy, obesity, periodontitis, inflammatory disorders), and other relevant results. Other reviewers, M.R.G. and M.R. and M.S., solved any disagreements.

To address biological and analytical heterogeneity across studies, data were additionally charted according to: (i) biofluid source (plasma, serum, saliva, or other human-derived fluids), (ii) miRNA compartment (extracellular vesicle/exosomal vs circulating cell-free), and (iii) analytic platform (e.g., next-generation sequencing, qRT-PCR, GWAS-based approaches).

Given that different biofluids and miRNA compartments may reflect distinct biological processes and are not directly interchangeable, findings were interpreted descriptively within matrix type. No attempt was made to pool or directly compare miRNA expression patterns across different biological matrices.

## 3 Results

### 3.1 Study selection process

The search strategy identified 209 records: 122 from Web of Science, 67 from Pubmed, 17 from CINAHL, and 3 through reference screening. After removal of duplicates, 148 unique records remained for screening.

Title and abstract screening resulted in the exclusion of 124 records that did not meet predefined eligibility criteria. Full texts of 24 studies were subsequently assessed for eligibility.

Of these, 5 studies were excluded because they were based exclusively on animal models [[26], [27], [28], [29], [30]]. Five additional studies were excluded due to methodological characteristics that did not meet the inclusion criteria defined for human circulating or exosomal miRNA quantification within clearly characterized sleep phenotypes [[31], [32], [33], [34], [35]]. Four studies were excluded because sleep phenotyping or clinical context was not aligned with SSD, IS, or ISOSS definitions as required by the review protocol [18,[36], [37], [38]]. One study was excluded due to the implementation of an intervention not directly related to sleep exposure or outcome [39]. One study was excluded because reported outcomes were not relevant to the predefined review questions [40], and another study was excluded due to an ineligible study population [41].

**Fig. 1:**
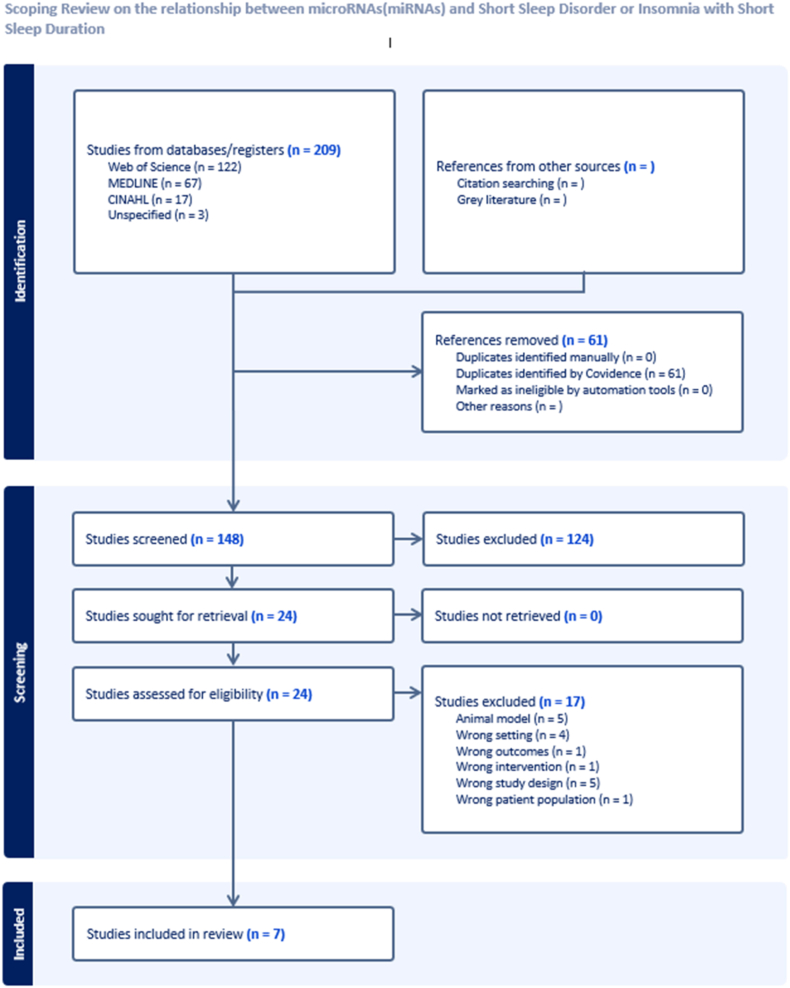
PRISMA flowchart illustrating the literature search and inclusion process.

A total of seven studies fulfilled all eligibility criteria and were included in the final scoping review. The study selection process is summarized in the PRISMA flow diagram (Fig. 1), and detailed reasons for exclusion at full-text level are presented.

These studies comprise Case-Control Studies [14,15,42] Cross-Sectional Studies [[43], [44], [45]] and Genome-Wide Association Studies (GWAS) [45,46]. A comprehensive summary of the selected studies is presented in Table 1. Detailed discussions of these studies will follow in the next paragraphs, following with the established review questions.

**Table 1:** Summary of included studies published in peer-reviewed journals until June 2025.

Author (Year)	Country	Study Design	Population: N Age mean ± SD /Species)	SSD/ISSD Diagnosis	Diagnostic Method	miRNA Involvement (Up/Down/None)	Other Pathologies Studied	Included (Yes/No)
Zhang et al. 2025 [45]	China	CS and GWAS	N = 40. a) Insomnia: N = 20 43.82 ± 15.87 y.o. f45%/m55% b) Controls: N = 20 41.50 ± 14.05 y.o. f60%/m40% Humans	Chronic Insomnia, characterized by difficulty in initiating and sustaining sleep or waking prematurely without the ability to return to sleep. PSQI; MEQ-SA; CGI; Q-LES-Q-SF	blood exosomal miRNA Illumina HiSeq 2500 platform, DESeq2; miRNAs determined Benjamini– Hochberg corrected p-values (q-values, false discovery rate), and QRT-PCR validation of miRNA sequence data.	51 miRNAs; 21 miRNAs up, and 30 down: insomnia vs- controls . miR-517a-3p Down: drug use in insomnia . miR-7-5p Down: drug use in insomnia . miR-3127-5p smoking . miR-4435 smoking . miR-411-5p Down > Age . miR-409-3p Down > Age . miR-182-5p-Down chronic insomnia . miR-451^a^ Down chronic insomnia	Depression (HAMD), and Anxiety (HAMA)	Yes
Ansarin 2024 [44]	Iran	CS	N = 557; n mir = 24: each group 4groups (S.D/BMI): a) N.S./N. W = 145 b) N.S/O. W = 140 c) S.S./N. W = 130 d) S. S/O. W = 142 24 of each group selected for miRNA. 35.7 ± 6.9 y.o. f49%/m51% Humans	Sleep diary <7 h/s=SS >7 h/s = NS	250 μL Blood 1-RNAisolation 2-cDNA to each mir with RT	. miR-33a -Down in B, C, D comp. A -Down D comp. C . miR-181d- Down in B, C, D comp. A -Down C comp. B . miR-132-3p -UP in B, C, D comp. A -Up D comp. B, C . miR-378a- None	Obesity (BMI)	Yes
Holm 2022 [46]	USA	Retrospective and animal Obs. and GWAS study	a) Screened for potential miRNAs-using A.I algorithm from “The miRNA body-map” b) n = 14 human neuroblastoma cell lines expressed Hcrt. c) rat model; miRNA and mRNA quantifications (n = 25) d) rat model HCRT1 peptide quantification (n = 15) e) zebrafish model (n = 96; 48:48) f) GWAS: UK Biobank Mir137 involved in human sleep?	No direct clinical diagnosis: sleep duration	1 A.I algorithm from “The miRNA body-map” 2 confirmation mir in rat model, zebrafish model and Human GWAS Study.	. mir137Down in SS miR-665 Down in SS (less important)	mir-137 related to schizophrenia miR-137 as an evolutionarily conserved regulator of Hcrt expression	
Hijmans 2019(55)	USA	CS	N = 24 44–62 y.o. a) N.S. = 12 f50%/m50% b) S.S. = 12 f58%/m42%	No clinical diagnosis: sleep duration = SPAQ	Plasma 1 RNAisolation 2 cDNA PCR and miRNA specific primers	. miR-125a -Down in SS comp to NS . mir-126 Down in SS comp to NS . miR-146a- Down in SS comp to NS . miR-34a, miR-92a, miR-145 miR-150-None	None	Yes
Saus 2010 [42]	Spain	Genetic C.C.	N = 700 a) M.D. = 359 Unipolar 217 Bipolar 142 57.65 ± 15.33 y.o. f64.9%/m35.1% b) Control = 341 39.76 ± 11.92 y.o. f41.1%/m58.9%	Insomnia = HAMD (items 4, 5 a).	1 S.A. pre-miRNAs (miR-132, miR-219-1, miR-183/96/182 cluster) and their mature forms conserved between mice and humans 2 RFX4, PHLPP, CLOCK and ADCY6 (target sites conserved in humans. 3 Expression analysis of miR-182 and miR-182∗ by qRT-PCR PCR 4 RT of the genomic regions of miR and target gene. 5 in silico analysis for targets miR- of the clock machinery	MD: sequence variants within the precursor of miR-182 Abnormal processing of pre-miR-182 in patients carrying the T allele of the rs76481776, mutation implicates mir-182 UP regulation. .2 changes in the pre-miR-96. . miR-132 None . miR-219-1 None . miR-183 None		Yes
Su-Jin Baek 2021 [15]	Republic Korea	CC and RCT	N = 179 NGS: Case:10; control = 10 1 validation study: Case:30; control:30 2 validation study: Case 29; control:30 ROC: case = 20; control = 20	PSQI	1 NGS: Search for P.S miRNA in study 2 QRT-PCR in two validation cohort 3 ROC and logistic regression for estimation of miRNA accuracy in P.S quality	miR-4433b-3p down miR-619-5p down	P.S. and breast cancer, autism, stroke, enriched pathways by miR-4433b-3p included inflammation and inflammatory bowel disease. P.S. and colorectal cancer Associated with circadian clock	yes
Xiao Li 2025 [14]	China	1 animal model 2 CC study	2-n = 15 (case n = 7; Controls n = 8)	S.P.	exosomes purified from the plasma qRT-PCR	miR-182-5p: down	atherogenesis	yes

Tab.1- Abbreviations: A.I. = Artificial Intelligence; BMI= Body Mass Index; C.C. = Case-Control Study; C.S. = Cross-Sectional Study; CGI = Clinical Global Impression Scale; Comp = Compared; GWAS= Genome-Wide Association Study; GWmiREA = Genome-Wide miRNA Expression Analysis; HAM-D = Hamilton Depression Rating Scale; Hcrt = Hypocretin/Orexin; MD = Major Depression; MEQ-SA= Morningness-Eveningness Questionnaire Self-Assessment Version; m = Male; f = Female; NGS = serum miRNA sequencing N.W. = Normal Weight; O.W. = Obese Weight; Obs. = Observational; PCR= Polymerase Chain Reaction; P.S. = poor sleep; PSQI= Pittsburgh Sleep Quality Index; Q-LES-Q-SF = Quality-of-Life Enjoyment and Satisfaction Questionnaire; RNA = Ribonucleic Acid; S.P = sleep deprivation.

### 3.2 Primary questions

**Fig. 2:**
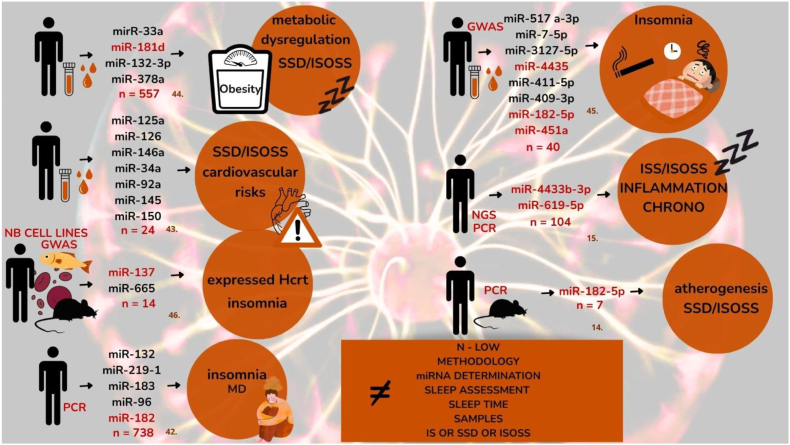
Differential miRNA expression across insomnia and short sleep phenotypes. Schematic summary of the studies included in this scoping review, illustrating altered microRNA (miRNA) profiles in insomnia (IS), short sleep duration (SSD), and insomnia with objective short sleep (ISOSS). Reported miRNAs are grouped according to their association with metabolic dysregulation, cardiovascular risk, inflammatory pathways, circadian regulation, and atherogenesis. Studies used heterogeneous methodologies, including GWAS, next-generation sequencing (NGS), and PCR-based approaches, across different biological matrices (whole blood, plasma, exosomes) and varying sleep assessment methods (objective and subjective). This methodological variability limits cross-study comparability and prevents identification of a unified miRNA signature. Abbreviations: IS = insomnia; SSD = short sleep duration; ISOSS = insomnia with objective short sleep; GWAS = genome-wide association study; NGS = next-generation sequencing; PCR = polymerase chain reaction.

**Interpretive note.** Across included studies, short sleep phenotypes were defined using heterogeneous measurement modalities and thresholds. Some studies operationalized short sleep using objective measures (actigraphy/PSG), whereas others relied on questionnaire-derived or self-reported sleep duration (e.g., PSQI-related items). Therefore, we report definitions as provided by each study and interpret findings within measurement modality, avoiding direct cross-study comparisons where cut-offs and assessment methods are not equivalent (Table 2, Fig. 2).

**Table 2:** MicroRNA and sleep.

Study	miRNA	Methods	Up	Down	None	Sleep disorder	Short Sleep	Normal Sleep
Ansarin 2024 [44]	miR-33a	transcription and qRT-PCR of miRNAs	X	X	X	SSD/ISOSS	X and Ob	Ss/Ns
	miR-181d	and qRT-PCR		X			X and Ob	Ss/Ns
	miR-132-3p						x and Ob	Ss/Ns
	miR-378a							Ss/Ns
Hijmans 2019 [43]	miR-125a	RT-PCR		X	X	SSD/ISOSS	X	Ss/Ns
	mir-126			X	X		X	Ss/Ns
	miR-146a			X	X		X	Ss/Ns
	miR-34a, miR-92a, miR-145 miR-150				X			Ss/Ns
								Ss/Ns
								Ss/Ns
								Ss/Ns
Holm 2022 [46]	mir137	GWAS		X		No sleep assessment IS		
	miR-665			X				
Saus 2010 [42]	miR-132	qRT-PCR	X		X	Late insomnia		
	miR-219-1				X			
	miR183				X			
	mir96				2changes?			
	mir182 cluster)							
Zhang 2025 [45]	miR-517a-3p	GWAS	21mirna?	Thirty mir		Chronic Insomnia		
	miR-7-5p			X		Insomnia and drug		
	miR-3127-5p			X		Insomnia and drug		
	miR-4435			X		Smoking		
	miR-411-5p			X		Smoking		
	miR-409-3p			X		Age		
	miR-182-5p			X		Age		
	miR-451a					Chronic Insomnia		
						Chronic Insomnia		
Su-Jin Baek 2021 [15]	miR-4433b-3p	1-NGS		X		Poor Sleep	X	
	miR-619-5p	2,3- qRT-PCR		X			X	
		4-ROC						
Xiao Li 2025 [14]	miR-182-5p:	qRT-PCR		X		Atherogenesis, SSD	X	

IS:Insomnia GWAS: Genome Wide Association Study; Ob: Obese; Ns: Normal Sleep; SS: Short Sleep; RT-PCR: Real-Time Polymerase Chain Reaction.

#### 3.2.1 How is literature addressing the epigenetic modifications of miRNA in SSD or in ISOSS?

Four studies accessed miRNA levels in patients with SSD and ISOSS [14,15,43,44]. Xiao Li et al. [14] examined the effects of sleep deprivation on human circulating exosomes (sample size n = 7; control group n = 8), finding a significant downregulation of miR-182-5p. Their results showed that inhibition of miR-182-5p led to a marked decrease in cell viability, an increase in cell apoptosis rates, and an elevated release of pro-inflammatory cytokines, particularly IL-18 and IL-1β, in comparison to control groups. Conversely, augmentation of miR-182-5p counteracted the pro-inflammatory and pro-apoptotic effects associated with exosomes derived from sleep-deprived conditions. Baek et al. [15] utilized Next-Generation Sequencing (NGS) for miRNA extraction (sample size n = 5; control group n = 5), followed by polymerase chain reaction (PCR) validation (sample size n = 30; control group n = 30), a subsequent randomized controlled trial (RCT) validation study (sample size n = 49; control group n = 50), and receiver operating characteristic (ROC) analyses (sample size n = 20; control group n = 20). Their research found statistically significant alterations in the expression of miR-4433b-3p and miR-619-5p, both of which were downregulated in subjects with poor sleep quality. miR-4433b-3p was linked to inflammatory pathways. Additionally, two genes, GBN5 and TP53, had correlation with the expression levels of miR-619-5p. Both GBN5 and TP53 are implicated in circadian rhythm regulation and the modulation of the circadian clock in response to photoperiod changes.

Ansarin and coworkers [44] employed polymerase chain reaction (PCR) techniques to determine that hsa-miR-33a expression was reduced in participants who experienced SSD, irrespective of their weight status, encompassing both obese and non-obese cohorts. This reduction in expression was more pronounced in individuals classified as obese who also experienced short sleep. Similarly, hsa-miR-132-3 showed a comparable pattern, showing a potential association between these microRNAs, SSD, and ISOSS within the context of obesity. However, in contrast, hsa-miR-132-3 expression was elevated among groups with SSD/ISOSS and obesity, with the peak expression seen in participants manifesting both conditions simultaneously. The expression levels of hsa-miR-181d were also diminished in participants with SSD/ISOSS, with a more substantial reduction noted in those of normal weight experiencing short sleep. In contrast, no significant changes were seen in the expression of hsa-miR-378a across any of the studied groups.

The investigation conducted by Hijmans et al. [43] explored the correlation between inadequate sleep and its consequent effects on circulating microRNA profiles. This study involved participants with normative sleep durations (n = 12; 7–9 h per night) and individuals characterized as short sleepers (n = 12; 5–6.8 h per night). The results revealed a significant downregulation of specific microRNAs, including miR-125a, miR-126, and miR-146a, within the short sleeper cohort. This finding suggests a potential pro-atherogenic profile associated with insufficient sleep. Nonetheless, there were no notable alterations in other circulating microRNAs examined. Furthermore, the analysis did not discern significant variances in the circulating levels of miR-34a, miR-92a, miR-145, and miR-150 between the groups, showing that not all vascular-related microRNAs are equally subject to the influence of sleep duration.

Holm et al. [46] studied the evolutionarily conserved microRNA, miR-137, and its regulatory role on the neuropeptide hypocretin/orexin (HCRT), as well as its later effects on sleep dynamics. This research integrated a multifaceted method, including retrospective analyses, animal observational models, and a GWAS study to elucidate the relationship between miR-137 and sleep regulation. The findings of the study highlight miR-137 as a regulator of HCRT expression, showing its potential significance in elucidating the pathophysiology of sleep disorders, such as IS. However, the study does not include a comprehensive assessment of sleep-related phenotypes, nor does it categorize ISOSS or SSD.

Saus et al. [42] conducted an observational case-control study to investigate the association between genetic variants of pre-miR-182 and the dysregulation of circadian rhythms in patients diagnosed with major depression (MD) presenting with late IS. In this study, a sleep assessment was conducted utilising items 4, 5, and 6 of the Hamilton Depression Rating Scale (HAM-D), categorising patients based on IS type: early (n = 253), middle (n = 236), and late insomnia (n = 249), however, IS was not characterized in SSD or ISOSS clusters.

The findings indicate that the identified sequence variants within pre-miR-182 contribute to the abnormal processing of the precursor miRNA in individuals with MD. Specifically, the presence of the T allele of the rs76481776 polymorphism was correlated with disrupted circadian rhythms and an elevated risk of late IS among affected patients. This polymorphism may be linked to the overexpression of miR-182, resulting in the downregulation of its target genes, which include essential components of the circadian clock. Zhang et al. [45] also conducted a comprehensive observational case-control study to elucidate the role of blood exosome-derived miRNA dysregulation in chronic IS, without characterizing IS in SSD or ISOSS clusters, The study identified a total of 51 modulated miRNAs, with 21 being upregulated and 30 downregulated in the insomnia group compared to controls. Among the salient findings, miR-517a-3p and miR-7-5p appear to have negative associations with pharmaceutical drug use in IS patients.

Cumulatively, the data show that miR-182-5p and miR-451a could serve as potential predictors for chronic IS, with significant downregulation seen in serum exosomes of the IS cohort. Specifically, hsa-miR-451a and hsa-miR-182-5p achieved sensitivity rates of 0.8 and specificity rates of 0.9 in predicting chronic IS patients. Importantly, hsa-miR-20a-5p levels did not show significant differences between groups.

#### 3.2.2 Does the composition (presence, diversity, and relative abundance) of miRNA in SSD or in ISOSS differ from the miRNA in individuals without sleep disturbances or those with insomnia but no short sleep duration?

Xiao Li et al. [14], Baek et al. [15], Ansarin et al. [44], and Hijmans et al. [43] studied miRNA expression in normal sleepers versus those with SSD and ISOSS, but small sample sizes and varied methods may affect their conclusions. Xiao Li et al. [14] found reduced miR-182-5p in sleep-deprived subjects, notably in the intestine, muscle, and aorta. Su-Jin Baek [15] used NGS and RCT to show miR-619-5p, miR-642, miR-1273d, and miR-4433b-3p were downregulated in poor sleepers. Ansarin et al. [44] noted miR-33a and miR-181d downregulation in obese short sleepers, with less miR-181 change in ISOSS, but *only* 96 subjects limited power. Hijmans et al. [43] found lower miR-125a, miR-126, and miR-146a in short sleepers, with just 24 participants. Zhang et al. [45] reported miR-182-5p and miR-451a downregulation in IS patients, suggesting them as markers for chronic IS, echoing other findings in animal models and human validation [14]. Saus et al. [42] found that abnormal pre-miR-182 processing in IS patients with the T allele of rs76481776 may lead to circadian rhythm issues, increasing late IS risk in MD patients. Holm et al. [46] noted a potential miR-137 and IS link but did not provide IS, SSD or assessment methods.

#### 3.2.3 Is there sufficient evidence that cardiometabolic comorbidities associated with patients with IS, SSD and ISOSS imply miRNA alterations?

In the study conducted by Xiao Li et al. [14], the findings may suggest that plasma exosomes derived from sleep-deprived individuals exert pro-inflammatory and pro-apoptotic effects on endothelial cells subjected to oxidative low-density lipoprotein (ox-LDL) or lipopolysaccharide (LPS) injury, as compared to control exosomes.

The analysis of downregulated miRNAs within endothelial cells exposed to the ox-LDL-induced inflammatory model revealed that miR-182-5p might be a particular candidate of miR-182-5p resulted in a decrease in cell viability, an increase in cell apoptosis, and heightened secretion of pro-inflammatory cytokines, specifically IL-18 and IL-1β, in comparison to control groups. These observations could show a link between chronic sleep deprivation, the downregulation of miR-182-5p, and the ensuing pro-inflammatory and pro-apoptotic responses in endothelial cells.

The results of the study conducted by Hijmans et al. [43] write down a marked downregulation of miR-125a, miR-126, and miR-146a in individuals classified as short sleepers. The identified miRNAs are known to be integral to the regulation of endothelial function, and their diminished expression may suggest a compromise in vascular integrity and an elevation in inflammatory processes. The implications of this downregulation are substantial; a reduction in these protective miRNAs may act to enhance susceptibility to atherogenesis, thereby increasing the cardiovascular risk profile of affected individuals.

### 3.3 Secondary questions

#### 3.3.1 Is there a potential association between specific miRNA and the severity of SSD or ISOSS symptoms?

At present, the scarcity of empirical evidence makes it challenging to definitively conclude the extent to which specific miRNAs are correlated with the severity of SSD or ISOSS symptoms. While it is plausible that such a connection might exist, the underlying mechanisms driving any potential relationship remain unexplored. The hypothesis suggesting a link between miRNA expression levels and the manifestation of these sleep disorders requires more comprehensive investigation.

#### 3.3.2 Is there sufficient evidence that SSD or ISOSS treatment could affect miRNA quantity or quality over time?

The current body of literature investigating the effects of treatment for SSD and ISOSS on the quantity and quality of miRNAs is limited. While some studies have found a relation between specific miRNAs and the pathophysiology of these sleep disorders, the evidence remains scarse, characterised by heterogeneous methodologies and a lack of rigorous validation. Consequently, the hypothesis that treatment interventions for SSD and ISOSS may influence miRNA expression patterns over time is plausible, yet currently insufficiently substantiated.

Table 2 shows a summary of the results obtained in the assorted studies evaluated in this ScR.

## 4 Discussion

### 4.1 How is literature addressing the epigenetic modifications of miRNA in SSD or in ISOSS?

The sample sizes considered in the various studies are limited, each study employs distinct methodological frameworks tailored to their specific research objectives, leading to inconsistencies in the data collected and analyzed. The starting samples from which miRNAs are isolated are not uniform, contributing to variability in the results.

An additional source of heterogeneity arises from the biological matrix and miRNA compartment analyzed across studies. Circulating cell-free miRNAs and extracellular vesicle (exosomal) miRNAs may reflect distinct biological processes, including passive release from damaged cells versus active intercellular signaling mechanisms. Furthermore, plasma, serum, and saliva differ in protein composition, vesicle content, and susceptibility to pre-analytical variability.

These biological differences limit direct cross-study comparability and complicate attempts to define a unified miRNA signature associated with SSD or ISOSS. Future research should prioritize matrix standardization and clearly distinguish exosomal from non-vesicular miRNA fractions to improve reproducibility and interpretability.

Not all studies categorically delineate insomnia into clusters such as Short Sleep Disorder (SSD) and Insomnia Objective Short Sleep (ISOSS), which further obscures the understanding of these conditions. The definition of “short sleep” varies among studies, with differing criteria regarding the duration considered as short sleep, complicating the synthesis of data and interpretation of results. Importantly, this heterogeneity is not limited to cut-offs but extends to the underlying measurement modality. Objective short sleep duration (actigraphy/PSG) and questionnaire-derived or self-reported short sleep reflect related but non-equivalent constructs, with different susceptibility to recall bias, misperception, and context effects. For this reason, we preserved each study's operational definition and interpreted miRNA findings within the same measurement modality rather than pooling across subjective and objective definitions. This limitation substantially reduces comparability across studies and precludes generalizable conclusions regarding a unified miRNA signature of SSD/ISOSS.

Only a few selected studies consider the phenomenon of short sleep [14,15,43,44]. In contrast, the research conducted by Zhang [45] and Saus [42] does not subdivide insomnia into the short sleep clusters. Furthermore, the study by Holm [46] notably lacks a comprehensive sleep assessment.

Although Ansarin's study [44] incorporates short sleep, only two of the groups examine this aspect: one group includes individuals with normal weight and short sleep, while another encompasses those with obesity and short sleep. This could potentially introduce bias in the findings about miRNAs. Within the cohort of normal-weight individuals experiencing short sleep, which consists of 130 patients, only 24 participants are studied for miRNA expression, and no rationale is provided for the selection of these specific individuals. Notably, hsa-miR-181d exhibited a more pronounced downregulation among individuals with SSD/ISOSS and normal weight compared to those in the obese group with normal sleep patterns. This finding is notable because it reduces the potential confounding effect of obesity inducing miRNAs alterations.

Hijmans's [43] research includes a cohort of just 12 patients, focusing on miRNAs associated with atherosclerosis. The findings show that circulating levels of miR-34a and miR-92a are elevated, while miR-125a, miR-126, miR-145, miR-146a, and miR-150 are diminished in middle-aged adults who experience short sleep. Conversely, Baek's [15] study is divided into four phases, with varying sample sizes: 5 individuals with short sleep in Phase 1, 30 in Phase 2, 49 in Phase 3, and 20 in Phase 4. Xiao Li's [14] investigation is limited to only 7 patients diagnosed with SSD/ISOSS.

Zhang's [45] study does not address clustered classifications but rather focuses on chronic insomnia, presenting a sample size of 20. Holm's [46]research involves 14 neuroblastoma cell lines, while Saus's [42] work includes a large cohort of 738 participants; however, in this study, insomnia is categorised into chronic, mild, moderate, and severe classifications rather than the SSD/ISOSS clusters.

The criteria used to define short sleep are also heterogeneous across studies. Ansarin [44] defines short sleep as a duration of less than 7 h, while Hijmans [43] finds it as falling within the range of 5.0 to 6.8 h per night. Holm's study [46], however, provides no explicit sleep assessment, referencing only sleep duration data derived from the UK Biobank. Saus [42] categorises insomnia into early, middle, and late stages, with respective sample sizes of 253, 236, and 249 participants, while Zhang [45] classifies patients as experiencing chronic insomnia. Baek [15]evaluates poor sleep quality through the Pittsburgh Sleep Quality Index (PSQI), and Xiao Li's [14] criteria focus on participants engaged in night shift work for over one year, targeting those who have worked a minimum of 3 h between midnight and 5:00 a.m., at least three nights per month, alongside eight female control participants.

Methodologically, Ansarin's [44] study aims to investigate the relationship between SSD/ISOSS, obesity, and miRNAs, employing RNA isolation and synthesising complementary DNA (cDNA) specific to each miRNA of interest using a reverse transcription (RT) reaction. In contrast, Hijmans [43] adopts distinct techniques, using specific miRNA probes and employing the miScript Reverse Transcription Kit for RNA isolation, after performing PCR amplification with the miScript SYBR Green PCR kit.

Holm et al. [46] screened for potential miRNAs targeting human hypocretin using four predictive algorithms (MicroCosm, TargetScan, DIANA, and PITA) as part of “The miRNA body-map,” finding several candidates including hsa-miR-137, hsa-miR-637, and hsa-miR-654-5p. The authors find miR-137 as a pivotal regulator of HCRT expression, highlighting its potential ramifications for the understanding of sleep disorders, including IS. Saus [42] confirmed the conservation of five different pre-miRNAs (miR-132, miR-219-1, and the miR-183/96/182 cluster) between murine and human models, employing qRT-PCR for expression analysis and targeted resequencing of genomic regions containing specific miRNAs and their corresponding target gene sites, alongside in silico evaluation for potential targets of miR-182 involved in circadian regulation.

Zhang's study [45] utilized blood exosomal miRNA and Illumina methods for identification, deciding significantly differentially expressed miRNAs (DE miRNAs) through corrected p-values and confirming the miRNA sequence data with qRT-PCR. Su-Jin Baek [15] applied next-generation sequencing (NGS) for miRNA identification, followed by confirmation using reverse transcription-polymerase chain reaction (RT-qPCR) and receiver operating characteristic (ROC) analysis. Xiao Li [14]implemented Western blot analysis, purifying and hybridising labelled RNA to microRNA arrays, with next analysis conducted using an Agilent microarray scanner.

The disparate methodologies and aims prevalent across these studies hinder the ability to draw coherent conclusions on the relationships between short sleep, insomnia, and associated miRNAs. The resulting variances underscore the pressing need for standardisation in research approaches to enable more meaningful comparisons and insights within the field. Xiao Li [14] and Zhang et al. [45] investigate the role of microRNA (miRNA), particularly miR-182-5p. It is pertinent to highlight that both studies emphasise the significance of microRNA-182-5p, supporting the work by Saus et al. [42]. Thus, these studies may provide insights into the molecular ramifications of sleep deprivation and elucidate the role of miR-182-5p in mediating its effects.

While the findings of Hijmans et al. [43] underscore the intricate relationship between insufficient sleep and alterations in circulating miRNA profiles, it is essential to acknowledge the limitations inherent in the study's method. Specifically, the cohort size appears small, which require cautious interpretation of the results.

Intriguingly, the expression of miR-4433b-3p demonstrated reproducibility in male participants, whilst both miR-4433b-3p and miR-619-5p exhibited consistent results in female participants [15]. Functional enrichment analysis of the network genes found fifteen pathways pertinent to “inflammation” and “circadian” processes, which could be related to poor sleep quality. miR-4433b-3p showed associations with inflammation-related pathways and inflammatory bowel disease pathways. Six genes—PPP1CB, PPP1CC, CREBBP, HELZ2, NCOA1, and TBL1X—linked to miR-619-5p for their relevance to circadian rhythm regulation. These findings could suggest that the dysregulation of miR-4433b-3p and miR-619-5p, alongside their target genes, may have critical implications for understanding the molecular mechanisms underlying poor sleep quality and its related pathways. Interestingly, in literature the miR-619-50 is usually associated with chronic pulmonary obstructive disease (COPD) [47], and different types of cancer like lung [48], colon [49], ovarian [50] or pancreatic cancer [51].

### 4.2 Does the composition (presence, diversity, and relative abundance) of miRNA in SSD or in ISOSS differ from the miRNA in individuals without sleep disturbances or those with insomnia but no short sleep duration?

Diverse miRNA profiles in studies on IS, SSD, and ISOSS suggest deeper investigation in this field. Unique miRNAs emerge for each condition, but a clear pattern is still elusive.

This discrepancy may be due to a plethora of factors, including methodological differences, sample sizes, population characteristics, and the specific contextual frameworks of each study. For instance, the delineation of IS as a unique construct, compared to alterations resulting from acute or chronic sleep deprivation, may lead to divergent miRNA expression profiles. Furthermore, the influence of confounding variables, such as age, sex, and underlying health conditions, could contribute to the heterogeneity seen in the findings.

Given these considerations, it is imperative that future studies encompass larger, more homogeneously defined populations, alongside standardized methodologies for the identification and quantification of miRNAs. Longitudinal studies examining changes in miRNA profiles in relation to sleep patterns and cardiovascular health over time may also be particularly beneficial. Establishing a clearer understanding of the miRNA landscape in these contexts could offer valuable insights into the underlying pathophysiological mechanisms and, later, inform the development of targeted therapeutic strategies.

### 4.3 Is there sufficient evidence that cardiometabolic comorbidities associated with patients with insomnia, ISOSS and SSD imply miRNA alterations?

Xiao Li et al. [14] conducted a study that revealed a significant increase in the release of pro-inflammatory cytokines, specifically IL-1β and IL-18, in human umbilical vein endothelial cells treated with exosomes derived from individuals engaged in night shift work. These findings could show that exosomes originating from sleep-deprived plasma exert pro-inflammatory and pro-apoptotic effects on endothelial cells compared to those from control exosomes.

In further elucidating the underlying mechanisms, the authors focused on four downregulated microRNAs (miRNAs) in this inflammatory context, finding miR-182-5p as a noteworthy candidate. The inhibition of miR-182-5p resulted in a marked reduction in cell viability, an increase in apoptosis, and an enhanced release of IL-18 and IL-1β compared to controls. miR-182-5p appears as a critical regulator of the pro-inflammatory effects induced by sleep deprivation-derived exosomes. Levels of both the mature form of miR-182-5p and its precursor, pre-miR-182-5p, showed significant reductions following an eight-week period of sleep deprivation across various tissues, including the intestine, skeletal muscle, and aorta.

hsa-miR-182-5p exhibited promising predictive capabilities for chronic IS, miR-182-5p demonstrated correlations with IS, MD, and anxiety. MiR-182-5p might play a pivotal role in modulating the inflammatory response via its regulatory effects on target genes associated with immune function. Research has indicated that miR-182-5p can influence the activation of macrophages, promoting a pro-inflammatory phenotype through the inhibition of anti-inflammatory mediators [52]. The dysregulation of miR-182-5p has been implicated in various pathological conditions, including atherosclerosis, where it promotes vascular inflammation and plaque instability [53]. The literature suggests that miR-4433b-3p, as referenced in the study by Baek et al., may be associated with the diagnosis of cerebrovascular disorders through the application of brain imaging techniques [54]. Complementary research by Hijmans (2019) [43] assessed the influence of habitual short sleep on specific vascular-related miRNAs. The results indicate a potential elevation in circulating miR-34a and miR-92a, while levels of miR-125a, miR-126, miR-145, miR-146a, and miR-150 were significantly lower in middle-aged adults experiencing short sleep. The observed downregulation of miR-125a, miR-126, and miR-146a signifies a potential impairment in biological pathways related to vascular health and inflammatory responses. These miRNAs documented for their crucial roles in endothelial function; thus, their decreased expression may correlate with an increased risk of atherogenesis among individuals with inadequate sleep. However, the studies do not present convergent evidence about the identified microRNAs, showing the necessity for further research involving larger cohorts to yield more definitive and consistent results. The current findings, while valuable, are limited by sample sizes and the potential variability inherent in smaller studies. Thus, a more extensive investigation is called for to corroborate the implications of these microRNAs and to elucidate their roles in the context of sleep deprivation and its associated inflammatory responses. Retaining a diverse and adequately powered sample could enhance the reliability of the data and offer clearer insights into the biological mechanisms at play, thereby contributing to a more comprehensive understanding of the relationship between sleep and cardiovascular health.

### 4.4 Secondary questions

#### 4.4.1 Is there a potential association between specific miRNA and the severity IS or SSD or ISOSS symptoms?

Future studies that aim to evaluate the expression profiles of specific miRNAs in individuals diagnosed with IS, SSD and ISOSS, alongside clinical assessments of symptom severity, are essential to elucidate these potential associations. The development of a robust understanding of how miRNAs may influence or reflect the severity of sleep disorder symptoms could pave the way for novel diagnostic tools and therapeutic strategies targeting the underlying pathophysiology of these conditions.

#### 4.4.2 Is there sufficient evidence that SSD or ISOSS treatment could affect miRNA quantity or quality over time?

To advance the field, longitudinal studies employing standardised methodologies are imperative, enabling the investigation of changes in miRNA expression pre- and post-treatment for SSD and ISSD. Such studies would facilitate a more comprehensive understanding of whether treatment can elicit significant alterations in miRNA quantity or quality over time.

While it is reasonable to assume a correlation between IS, SSD or ISOSS treatment and miRNA dynamics, the current evidence is insufficient to support this assertion robustly. Future research efforts are essential to elucidate the potential impacts of therapeutic interventions on miRNA profiles associated with sleep disorders, contributing to the development of more effective diagnostic and therapeutic strategies.

## 5 Conclusions

The current scoping review offers significant insights into the research surrounding miRNAs, particularly in elucidating the relationships between IS, SSD and ISOSS with a range of miRNAs, including miR-182-5p, miR-4433b-3p, miR-619-5p, miR-33a, miR-181d, miR-132-3p, miR-125a, miR-126, miR-146a, miR-34a, miR-92a, miR-145, miR-150, miR-137, miR-665, miR-132, miR-219-1, miR-183, miR-96, miR-517a-3p, and miR-7-5p. Nonetheless, it is important to acknowledge that these findings call for further validation through later research.

The current literature reflects a limited scope of investigations on the interplay between miRNAs, IS, SSD, ISOSS, and their cardiometabolic relationships. Consequently, there is a pressing need for expanded research aimed at delineating the potential correlations among these variables. Such studies should focus on characterising miRNA alterations by stratifying patient samples based on the severity of symptoms and the degree of sleep impairment.

This approach may enhance our understanding of the intricate relationships between short sleep duration, insomnia, and miRNA expression and inform the development of novel therapeutic interventions.

## CRediT authorship contribution statement

**Susana Perdigoto:** Writing – original draft, Visualization, Validation, Supervision, Resources, Project administration, Methodology, Investigation, Formal analysis, Data curation, Conceptualization. **Miguel Meira e Cruz:** Writing – review & editing, Writing – original draft, Visualization, Validation, Supervision, Resources, Project administration, Methodology, Investigation, Conceptualization. **Manuel Remesal:** Writing – review & editing, Visualization, Validation, Supervision. **Mauro Scala:** Visualization, Validation, Methodology. **María del Rocío González Soltero:** Writing – review & editing, Visualization, Validation. **Clara Azpeleta Noriega:** Writing – review & editing, Visualization, Data curation. **Miguel de Pedro:** Writing – review & editing, Writing – original draft, Visualization, Validation, Supervision, Resources, Project administration, Methodology, Investigation, Formal analysis, Data curation, Conceptualization.

## Declaration of competing interest

The authors declare that they have no known competing financial interests or personal relationships that could have appeared to influence the work reported in this paper.
